# The biodiversity of *Lactobacillus* spp. from Iranian raw milk Motal cheese and antibacterial evaluation based on bacteriocin-encoding genes

**DOI:** 10.1186/s13568-017-0474-2

**Published:** 2017-09-18

**Authors:** Fahimeh Azizi, Mohammad B. Habibi Najafi, Mohammad R. Edalatian Dovom

**Affiliations:** Department of Food Science and Technology, Faculty of Agriculture, Ferdowsi University of Mashhad, Azadi Square, Mashhad, Iran

**Keywords:** Antimicrobial activity, Bacteriocin, Biphasic approach, Cheese, *Lactobacillus*, Traditional dairy products

## Abstract

*Lactobacilli*, as the largest group of lactic acid bacteria, produce large amounts of antimicrobial metabolites such as organic acids, fatty acids, ammonia, hydrogen peroxide, diacetyl and bacteriocin, which inhibit the growth of pathogenic bacteria and increase shelf life of food. The aim of this study was to identify the *Lactobacillus* spp. isolated from Iranian raw milk Motal cheese and to detect the presence of bacteriocin genes in the isolated *Lactobacillus* strains exhibiting antimicrobial activity. For this purpose, 6 Motal cheese samples from Dasht-e-Moghan region, Iran, were subjected to microbial characterization. Nineteen *Lactobacillus* spp. were isolated and subsequently identified based on biochemical and molecular methods. According to the sequencing of isolates, *Lactobacillus* spp. consisted primarily of *Lactobacillus brevis*, *Lactobacillus plantarum*, *Lactobacillus casei* and *Lactobacillus buchneri*. The identified isolates were then evaluated for antimicrobial activity against *Escherichia coli* ATCC 25922, *Listeria innocua* ATCC 33090 and *Staphylococcus aureus* ATCC 25923. The results of PCR analysis using specific primers of genes encoding Bacteriocin, revealed the presence of Plantaricin A and Plantaricin EF in all *Lactobacillus plantarum* isolates and Brevicin 174A in 5 of *Lactobacillus brevis* isolates, whereas the gene encoding Pediocin PA-1 was not observed in any of examined isolates. It is therefore concluded that bacteriocinogenic isolates could be recommended as suitable candidates to be used as starter, adjunct-starter or antimicrobial agents for production of fermented and non-fermented products.

## Introduction

Conversion of carbohydrate to lactic acid by lactic acid bacteria (LAB) may be considered as the most important fermentation in food industry. The characteristic aroma, flavor, and texture of fermented foods (e.g., dairy, meat, and vegetables) are often due to growth of these bacteria. (Habibi-Najafi and Lee 2007; Hayaloglu et al. 2002; Wada et al. 2009). Some strains of LAB isolated from dairy and other fermented products may contribute to the safety and quality of foods owing to possessing antimicrobial agents. The bactericidal effects of such agents on wide range of pathogens such as *Listeria* monocytogenes, *Escherichia coli* and *Staphylococcus aureus* have been studied (Cintas et al. 2001; De Vuyst and Leroy 2007). On the other hand, some strains of LAB play a vital role in the digestive tract by producing antimicrobial metabolites such as bacteriocins and prevent the growth of pathogenic and infection microorganisms (Castro et al. 2011; Ghanbari et al. 2013; Parada et al. 2007; Ahmed et al. 2013; Mahrous et al. 2013). Bacteriocins are divided into two main classes, lantibiotic such as Nisin (class I), and nonlantibiotic such as Pediocin and PlantaricinEF (class II) (Noda et al. 2015).

In recent years, bacteriocins produced by LAB have been considered as food bio-preservatives due to the fact that LAB are among those microorganisms which generally regarded as safe (GRAS) (Leroy and De Vuyst 2004; Molloy et al. 2011; Verma et al. 2014).

Genome sequencing plays an important role in the accurate identification of bacteriocinogenic LAB (Ortolani et al. 2010). The genes encoding bacteriocin are located in operon clusters, which may be placed on the chromosome (such as PlantaricinST31), or plasmids (such as Plantaricin423) or transposons (such as Nisin A) (Knoll et al. 2008; Todorov 2009). Up to now, different types of bacteriocin locus such as Plantaricin, Pediocin PA-1/AcH, Sakacin674, SakacinP and BavaricinA have been identified from different strains of *L. plantarum* (C11, WCFS1, NC8, J23 and J51), *L*. *plantarum* WHE 92, *L*. *sake* 674, *L*. *sake* LTH673 and *L*. *bavaricus* MI401, respectively (Remiger et al. 1996; Xie et al. 2011).

Different types of cheese have been produced traditionally from various sources of milk in Iran. Traditional Motal cheese is produced locally in Dasht-e-Moghan from raw sheep’s milk without adding any starter culture. This type of cheese contained high fat and salt with friable texture. The aging and ripening occurs inside the sheep skin (Veljovic et al. 2007). In regards to the increasing demand for selection of microbial strains with protective properties, the aim of this study was to identify the *Lactobacillus* spp. isolated from Iranian raw milk Motal cheese and to detect the presence of genes encoding Plantaricin A, Plantaricin EF, Pediocin PA-1 and Brevicin 174A in the isolated *Lactobacillus* strains exhibiting antimicrobial activity to be used for commercial production as well as improvement the quality and safety of fermented food products,

## Materials and methods

### Sample collection

Six samples of traditional Motal cheese were collected from rural areas in Dasht-e-Moghan, Iran, and were immediately transported to the laboratory under refrigerated conditions for analysis.

### Microbiological analysis

Portions of 25 g of each sample were transferred under sterile conditions to stomacher bags and mixed with 225 ml sterile 2% (w/v) citrate sodium solution in a stomacher (Seaward Medical, London SE1 1PP, UK) for 5 min. Serial dilutions (10^−1^–10^−8^) were then prepared for each sample. For total count of bacteria, 1 ml of each dilution was pure plated in a plate containing PCA medium (Merck GmbH, Darmstadt, Germany) and was incubated at 35 °C for 48 h and the number of colonies on each plate with 30–300 colonies were counted. For enumeration of total LAB, 0.1 ml from each dilution was spread-plated on MRS medium (Merck GmbH, Darmstadt, Germany) and incubated at 37 °C in aerobic and anaerobic conditions. In order to provide anaerobic conditions, jars with Anaerocult type A gaspak were used (Merck GmbH, Darmstadt, Germany). Counting of total coliforms was performed on VRBA (Merck GmbH, Darmstadt, Germany), incubated for 24 h at 35 °C. The search for *Staphylococci* was viewed on BP medium containing Potassium Tellurite and egg yolk and incubation was done at 35 °C for 24–48 h (Antonsson et al. 2003; Şengül 2006; Turgay and Erbilir 2006; Guetouache and Guessas 2015).

### Isolation and phenotypic identification of LAB

To obtain pure cultures, colonies were randomly selected from the surface of each medium and streak plating was performed on the same medium (Guetouache and Guessas 2015). After implementation the gram reaction and catalase activity on isolates, only gram-positive and catalase-negative isolates were selected for further analysis by the following phenotypic tests: growth at 15 and 45 °C in MRS broth, growth at pH 4.4 and pH 9.6 in MRS broth, salt tolerance: growth with 6.5 and 18% NaCl in MRS broth and production of carbon dioxide from glucose by cultivation isolates in tubes with MRS containing a Durham’s tubes (Nikolic et al. 2008; Guetouache and Guessas 2015). All gram-positive and catalase-negative isolates were stored at −80 °C in MRS broth containing 15% glycerol (v/v).

### Molecular identification of isolates

#### DNA isolation

To extract bacterial genome, streak plating from each isolate on MRS agar was performed and then incubated at 37 °C for 48 h. A loop of grown colony was then transferred to MRS broth and incubated at 37 °C for 18 h for re-activation. Cells were then harvested, and the DNA extraction was performed with the GenElute™ Bacterial Genomic DNA kit (Thermo Fisher Scientific, Germany) according to the manufacturer’s protocol. Electrophoresis was performed on 1% agarose gel in TBE.1X buffer and photographed under UV light.

#### 16S rRNA sequencing

Primers 27F (5′-AGAGTTTGATYMTGGCTCAG-3′) and 1492R (5′- GGTTACCTTGTTACGACTT-3′) were used for amplification of the gene 16S rRNA (Alegría et al. 2009). Polymerase chain reactions (PCR) were carried out in a thermocycler (Eppendorf, Hamburg, Germany) in a total volume of 20 μl containing10 μl 2 × PCR master mix (Amplicon, Denmark), 1 ml of the mixed primers 27F and 1492R (concentration 10 pmol/μl), 8 μl nuclease free deionized water and 1 μl template DNA, running under the following temperature program: initial denaturation of DNA for 5 min at 95 °C, 35 cycles of 45 s at 94 °C, 50 s at 55 °C, and 2 min at 72 °C; and final extension for 10 min at 72 °C (Emerenini et al. 2013). 5 µl aliquots of the PCR products with 1 µl loading buffer were analyzed by electrophoresis using a 1% (w/v) agarose gel in Tris Boric acid EDTA (TBE. 1X) buffer at 100 V for 45 min. The gel was then placed in Gel doc (Cleaver scientific Ltd) to detect the presence a band of 1500 bp. The size of the DNA fragments was estimated using a GeneRuler 100 bp Plus DNA Ladder (Fermentase, Canada).

GenElute™ PCR clean-up column (Thermo Fisher Scientific, Germany) was used for purification before sending the extracted DNA for sequencing, according to the manufacturer’s instructions. An average of 850 bp nucleotides for each sequence from each side were read and compared with the databases using the BLAST program (http://blast.ncbi.nlm.nih.gov/Blast.cgi). Isolates with 98% or higher similarity in sequences were identified as the same species (Stackebrandt and Goebel 1994; Palys et al. 1997).

#### Phylogenetic tree construction

Sequence similarities of the studied *lactobacilli* isolates was predicted using Mega 5.1 software. The 16S rRNA sequences of isolates were multiple aligned using the ClustalW algorithm. Constructions were carried out using UPGMA (Unweighted Pair Group Method with Arithmetic Mean) method by means of taking 1000 as bootstrap value (Bunesova et al. 2014).

#### Detection of antibacterial activity

##### Agar spot test

Isolates were evaluated for production of antimicrobial agents against *Staphylococcus aureus* ATCC 25923, *Escherichia coli* ATCC 25922 and *Listeria innocua* ATCC 33090 as indicator microorganisms. To perform this test, 2.5 μl overnight cultures of each isolate was spotted on MRS agar and incubated anaerobically at 37 °C for 48 h. 10 µl of each indicator strain, grown in the BHI broth, was then added to 7 ml sterile soft agar (BHI broth + 0.75% agar). The inoculated soft agar with turbidity equivalent to 0.5 McFarland was poured onto the spotted MRS agar. These plates were incubated at 37 °C for 72 h. The presence of clear halo around the inoculated spot of each indicator bacteria lawn reflects the no growth of the indicator microorganism and thus the possessing of antimicrobial activity of examined isolate (Alegría et al. 2010; Al-Otaibi 2012).

##### Well diffusion agar test

In this method, the isolates that demonstrated antimicrobial properties were tested based on the presence of the clear halo. The inoculated cultures of the positive isolates in terms of antibacterial activity were centrifuged for 5 min at 5000×*g*. The pH of supernatant was measured and adjusted to neutral pH (6.5–7.0) with NaOH 1 N and was then filtrated by 0.45 μm pore size filter (Millipore, Bedford, MA, USA). Aliquots of 30 μl from 16 h cultures of indicator bacteria were diluted equivalent to 0.5 McFarland and were inoculated to 15 ml of BHI culture consisting of 1% molten agar. Aliquots of 30 μl filtered cell-free supernatant (CFS) of isolates were transferred to wells with a diameter of 6 mm, made with a sterile puncher. Plates were incubated for 48 h in 37 °C to investigate the antibacterial activity and halo formation (zone of growth inhibition). Isolates with clear zones of growth inhibition with a diameter more than 1 mm around wells were considered as positive (Alegría et al. 2010; Bettache et al. 2012).

#### Identification of genes encoding bacteriocin production

The identification was performed using PCR or molecular method. The specific primers of bacteriocin used in this study are shown in Table 1.

**Table 1:** Primers used throughout this study and their amplification details

Name	Sequence (5′ → 3′)	Size amplicon	Annealing temperature	References
Brevicin 174A-F	GTCTTAAATGCTAGGCTTGTCA	766	56	Noda et al. (2015)
Brevicin 174A-R	CTGGCAAGACAAACGGTTAG			
plnA-F	TAGAAATAATTCCTCCGTACTTC	573	55	Xie et al. (2011)
PlnA-R	ATTAGCGATGTAGTGTCATCCA			
plnEF-F	TATGAATTGAAAGGGTCCGT	516	54	Xie et al. (2011)
plnEF-R	GTTCCAAATAACATCATACAAGG			
Pediocin PA-1-F	AAAGATACTGCGTTGATAGG	1120	50	Xie et al. (2011)
Pediocin PA-1-R	GAGAAGCCATGCTGAAAG			

According to the bacteriocins sequences of *Lactobacillus brevis* 174 A (GenBank accession no. LC062087), *Lactobacillus plantarum* C11 (GenBank accession no. X9443) and *Pediococcus acidilactici* PAC1.0 (GenBank accession no. M83924), four sets of primers were designed for specific targets using CLC Main Workbench 5 and Primer Premier 5 software and prepared by Macrogen Company (Wada et al. 2009; Noda et al. 2015). After adding each component of the reaction mixture following the kit manufacturer’s instruction, microtubes were placed in the thermocycler and the temperature program was set according to the type of bacteriocin.

Amplification of plnA, plnEF and Pediocin PA-1 genes was performed under the following conditions: 95 °C for 5 min, followed by 30 cycles of denaturation at 94 °C for 30 s, annealing at different temperatures as shown in Table 1 for 1 min and extension at 72 °C for 1 min, and final extension at 72 °C for 10 min (Xie et al. 2011).

PCR of Brevicin 174A gene was conducted under the following conditions: 1 cycle of 5 min at 96 °C, 15 s at 58 °C and 30 s at 72 °C followed by 29 cycles of 1 min at 96 °C, 15 s at 58 °C and 30 s at 72 °C, and a 7 min final extension at 72 °C. The amplicons were purified using a purification kit (Thermo Fisher Scientific, Germany) and subjected to DNA sequencing (Bioneer, Korea) (Wada et al. 2009; Xie et al. 2011; Noda et al. 2015).

## Results

### Microbiological analysis

The mean microbial counts of all Motal cheese samples are reported in Table 2. The total average microbiota of all cheese samples was 14.13 × 10^4^ cfu g^− 1^. The microbiological analysis showed that the total average of *Staphylococcus aureus* and coliforms in six cheese samples was about 0.19 × 10^4^ and 0.13 × 10^4^ cfu g^−1^respectively. The average total counts of mesophilic LAB in aerobic and anaerobic conditions in all cheese samples was 6.6 × 10^4^ and 7.45 × 10^4^ cfu g^− 1^. Samples A and C showed the highest microbial population density on MRS (Table 2).

**Table 2:** Results of microbiological analysis of Motal cheese

Sample	Count (cfu g^− 1^)				
	Total count	LAB (aerobic)	LAB (anaerobic)	*S. aureus*	Coliforms
A	3 × 10^5^	5 × 10^4^	1 × 10^5^	ND	ND
B	2 × 10^5^	3 × 10^4^	1 × 10^5^	9 × 10^3^	2 × 10^3^
C	3 × 10^5^	3 × 10^5^	2 × 10^5^	2 × 10^3^	6 × 10^3^
D	3 × 10^3^	3 × 10^3^	5 × 10^3^	ND	ND
E	4 × 10^4^	1 × 10^4^	4 × 10^4^	5 × 10^2^	ND
F	5 × 10^3^	3 × 10^3^	2 × 10^3^	ND	ND

### Morphological and biochemical properties of *Lactobacillus* strains

A total of 64 colonies grown on MRS under aerobic and microaerophilic conditions at 37 °C were randomly isolated for further studies. The colonies were first morphologically observed under light microscope. Twenty-nine cocci and 35 rod were identified. Nineteen out of 35 rod-shaped gram-positive, and catalase negative bacteria were selected as *Lactobacilli* based on biochemical analysis (Table 3) (Pyar and Peh 2014). Fifteen isolates produced CO_2_ were considered heterofermentative and four strains as homofermentative.

**Table 3:** Physiological characteristics and preliminary identification of *Lactobacillus* spp. isolated from Motal cheese

Physiological tests	Preliminary identification of *Lactobacillus* spp.	
	+	−
Growth at 15 °C	19	0
Growth at 45 °C	0	19
Growth in medium with 6.5% NaCl	19	0
Growth in medium with 18% NaCl		19
Growth at pH 4.4	19	0
Growth at pH 9.6	0	19
Production of CO_2_ from glucose	15	4

+ Number of isolates with positive reaction; − number of isolates with negative reaction

### PCR amplification of the 16S rRNA gene

One of the general methods used to identify and differentiate LAB in dairy foods and products is the analysis of the 16S rRNA sequence (Zdolec and Filipović 2012). PCR using 27F and 1492R specific primers of 16S rRNA locus, produces amplicons in size of 1500 bp (results not shown). Nucleotide sequence of 16S rRNA is a suitable source for identification of bacteria isolates and phylogenetic tree construction (Kermanshahi and Peymanfar 2012). Table 4 illustrate *Lactobacillus brevis*, *Lactobacillus buchneri*, *Lactobacillus casei* and *Lactobacillus plantarum* species isolated form Motal cheese.

**Table 4:** Identities of pure isolates

Strain	Closest relative	Identity (%)	GenBank accession no.
M1	*Lactobacillus brevis*	99	KX572375
M2	*Lactobacillus brevis*	99	KX572376
M3	*Lactobacillus brevis*	99	KX572377
M4	*Lactobacillus brevis*	100	KX572378
M5	*Lactobacillus brevis*	100	KX572379
M6	*Lactobacillus brevis*	100	KX572380
M7	*Lactobacillus brevis*	99	KX572381
M8	*Lactobacillus brevis*	100	KX572382
M9	*Lactobacillus brevis*	99	KX572383
M10	*Lactobacillus brevis*	100	KX572384
M11	*Lactobacillus brevis*	99	KX572385
M12	*Lactobacillus brevis*	100	KX572386
M13	*Lactobacillus brevis*	100	KX572387
M14	*Lactobacillus buchneri*	99	KX572388
M15	*Lactobacillus casei*	100	KX572389
M16	*Lactobacillus plantarum*	100	KX572390
M17	*Lactobacillus plantarum*	100	KX572391
M18	*Lactobacillus plantarum*	99	KX572392
M19	*Lactobacillus plantarum*	100	KX572393

After cultivation, a varied set of isolates were detected using both phenotypic methods and PCR analysis of 16S rRNA (Randazzo et al. 2002). Despite being a small collection of isolated *Lactobacillus* species, *Lactobacillus brevis* species was the dominant species among the identified isolates using molecular methods in this collection.

### Phylogenetic tree construction of isolates

**Fig. 1:**
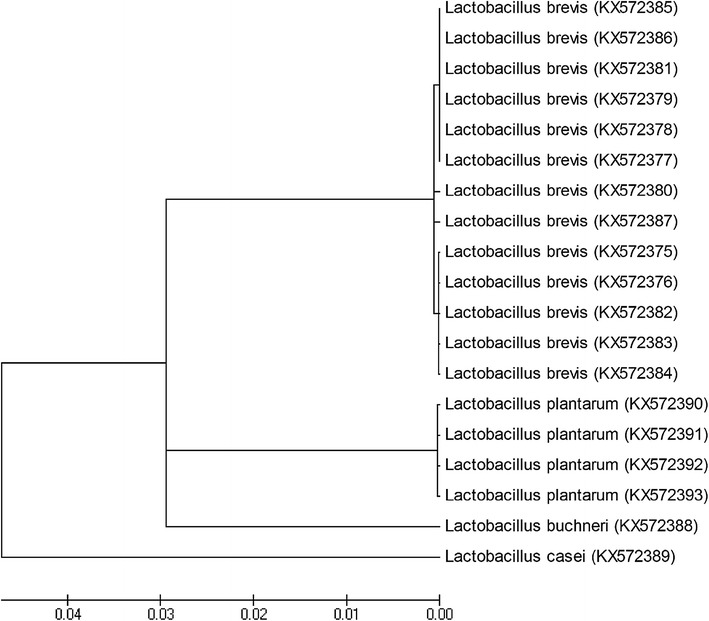
Phylogenetic tree based on 16S rRNA sequence analyses, showing the phylogenetic placement of representative strains isolated from Motal cheese

To infer the evolutionary relationship of identified *Lactobacillus* spp. a phylogenetic tree based on 16S rRNA sequence analyses was obtained using neighbor joining method (Fig. 1). The accession number for each sequence is placed between brackets.

In the phylogenetic tree built, all of the investigated isolates were well clustered into four batches and six sub-batches representing four species of identified *Lactobacillus*. These species comprised *Lactobacillus brevis*, *Lactobacillus plantarum*, *Lactobacillus buchneri* and *Lactobacillus casei* strains, respectively.

### Antibacterial activity

#### Agar spot assay

The effect of antimicrobial compounds produced by investigated isolates was performed in this method by spotted colonies on the bed of three indicator bacteria including *Staphylococcus aureus* ATCC 25923, *Listeria innocua* ATCC 33090, *Escherichia coli* ATCC 25922 in BHI medium (Alegría et al. 2010).

Based on the results presented in Table 5, indicator bacteria *Staphylococcus aureus* ATCC 25923, *Escherichia coli* ATCC 25922 and *Listeria innocua* ATCC 33090, were inhibited by 15, 5 and 10 isolates of *Lactobacillus* spp., respectively. These results indicated that *Staphylococcus aureus* ATCC 25923 was more sensitive to antimicrobial agents produced by examined isolates, compared to the other two indicators.

**Table 5:** Inhibition activity of isolated *Lactobacillus* genus isolates against indicators using the deferred spot assay

*Lactobacillus* species isolates	Indicator strain		
	*Staphylococcus aureus*	*Listeria innocoa*	*E. coli*
	ATCC25923	ATCC33090	ATCC 25922
*Lactobacillus brevis* M1	+^a^	−	(+)
*Lactobacillus brevis* M2	(+)	(+)	−
*Lactobacillus brevis* M3	−	−	−
*Lactobacillus brevis* M4	(+)	−	+
*Lactobacillus brevis* M5	(+)	−	−
*Lactobacillus brevis* M6	+	−	−
*Lactobacillus brevis* M7	+	−	(+)
*Lactobacillus brevis* M8	+	−	(+)
*Lactobacillus brevis* M9	−	−	(+)
*Lactobacillus brevis* M10	+	−	+
*Lactobacillus brevis* M11	(+)	−	−
*Lactobacillus brevis* M12	++	++	++
*Lactobacillus brevis* M13	−	−	−
*Lactobacillus buchneri* M14	(+)	−	−
*Lactobacillus casei* M15	−	−	−
*Lactobacillus plantarum* M16	+	−	−
*Lactobacillus plantarum* M17	++	++	(+)
*Lactobacillus plantarum* M18	++	++	++
*Lactobacillus plantarum* M19	++	++	++

^a^The number of crosses in the test is related to the diameter of the inhibition halo; in parenthesis, weak inhibition

#### Well diffusion assay

Based on the results shown in Table 6, *Staphylococcus aureus* ATCC 25923 and *Listeria innocua* ATCC 33090, were the most sensitive and resistant indicator bacteria to antimicrobial compounds produced by identified *Lactobacilli* isolates of Motal cheese.

**Table 6:** Antagonistic activity (well assay) of *Lactobacillus* genus isolates against indicator bacteria

*Lactobacillus* species isolates	The diameter of indicator bacteria		
	*Staphylococcus aureus* ATCC25923	*Listeria innocoa* ATCC33090	*E. coli* ATCC 25922
*Lactobacillus brevis* M1	1.7	–	1.1
*Lactobacillus brevis* M2	1.2	–	–
*Lactobacillus brevis* M4	–	–	–
*Lactobacillus brevis* M5	–	–	–
*Lactobacillus brevis* M6	1.1	–	–
*Lactobacillus brevis* M7	1.2	–	–
*Lactobacillus brevis* M8	1.7	–	–
*Lactobacillus brevis* M9	–	–	–
*Lactobacillus brevis* M10	2.8	–	1.5
*Lactobacillus brevis* M11	–	–	–
*Lactobacillus brevis* M12	3.3	2.9	4.5
*Lactobacillus buchneri* M14	–	–	–
*Lactobacillus plantarum* M16	1.7	–	–
*Lactobacillus plantarum* M17	3.4	2	1.2
*Lactobacillus plantarum* M18	4.5	3.2	4.3
*Lactobacillus plantarum* M19	6	3.7	4.8

#### Detection of bacteriocin structural genes

In order to detect the presence of genes encoding bacteriocin, PCR reactions were performed using four sets of specific primers; Plantaricin A, Plantaricin EF, Brevicin 174A and Pediocin PA-1. Products of 573, 596 and 766 bp were detected using specific primers of plnA, plnEF and bre174A, respectively. However, no DNA fragment was amplified using the specific primer of Pediocin PA-1 (Table 7).

**Table 7:** PCR amplification of bacteriocin genes from *Lactobacillus plantarum* and *Lactobacillus brevis* Motal cheese isolates

Bacteriocinogenic isolates	Bacteriocin gene			
	PlnA	PlnEF	Pediocin PA-1	Bre174A
*Lactobacillus plantarum* M16	+	+	−	
*Lactobacillus plantarum* M17	+	+	−	
*Lactobacillus plantarum* M18	+	+	−	
*Lactobacillus plantarum* M19	+	+	−	
*Lactobacillus brevis* M1				+
*Lactobacillus brevis* M2				
*Lactobacillus brevis* M6				
*Lactobacillus brevis* M7				+
*Lactobacillus brevis* M8				+
*Lactobacillus brevis* M10				+
*Lactobacillus brevis* M12				+

(+) gene present (−) no presence of gene, blank not performed PCR for determine that carried genes bacteriocin

**Fig. 2:**
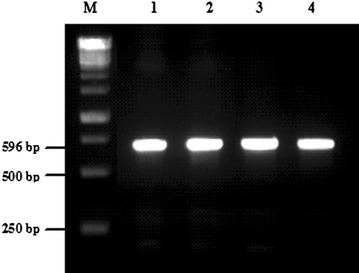
Detection of plnEF sequences in *Lactobacillus plantarum.* Detection of plnEF sequences in 4 *Lb. plantarum* isolates. Lane M: molecular weight marker 1kb; Lane 1: M16; Lane 2: M17; Lane 3: M18; Lane 4: M19

**Fig. 3:**
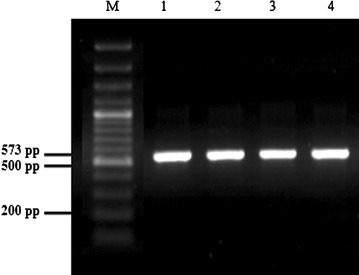
Detection of plnA sequences in *Lactobacillus plantarum.* Detection of plnA sequences in 4 *Lb. plantarum* isolates. Lane M: molecular weight marker 1kb; Lane 1: M16; Lane 2: M17; Lane 3: M18; Lane 4: M19

According to Fig. 2, plnEF gene was presented in *Lactobacillus plantarum*M16, M17, M18 and M19. Also, the performed PCR using plan primers in *Lactobacillus plantarum* M16, M17, M18 and M19 speices, showed the presence of plan bacteriocin in all of examined isolates (Fig. 3).

**Fig. 4:**
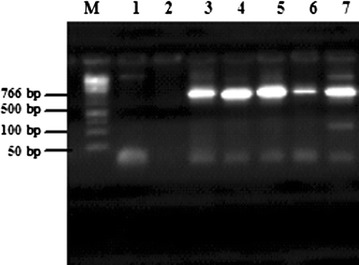
Detection of bre174A sequences in *Lactobacillus brevis.* Detection of bre174A sequences in 7 *Lb. brevis* isolates. Lane M: molecular weight marker 1kb; Lane 1: M2; Lane 2: M6; Lane 3: M1; Lane 4: M7; Lane 5: M8; Lane 6: M10; Lane 7: M12

The PCR results of bre174A specific primers revealed that among 7 *Lactobacillus brevis* subspecies examined for production of bacteriocins, 5 *Lactobacillus brevis* strains M1, M7, M8, M10, and M12 had bre174A bacteriocin gene (Fig. 4; Table 7).

#### Sequence and culture deposition

Sequences were submitted to the NCBI GenBank (http://www.ncbi.nlm.nih.gov/genbank) and acquired accession numbers of KX572375–KX572394 (Table 4). Isolates coded M8 and M12 were selected randomly and deposited into IBRC culture collection as IBRC- M11142, IBRC- M11141 respectively.

## Discussion

This study provides an overall analysis on *Lactobacillus* strains communities in Motal cheese. To the best of our knowledge, this is the first attempt performed on Motal cheese to isolate and identify *Lactobacillus* strains communities and detect the presence of bacteriocins in the identified strains. The mean value of LAB in Motal cheese was higher in comparison with other types of traditional raw milk products such as Klila cheese (Guetouache and Guessas 2015). *Lactobacilli* occurrence is usually higher in ripened semi-hard cheeses. This may also due to the differences in terms of milk quality as well as to the standardization of manufacturing facilities.

LAB species have been known the most frequent species in traditional raw milk cheeses which are affecting the quality of such category of cheeses (Mennane et al. 2007; Bautista-Gallego et al. 2014).

Based on phenotypic tests, the heterofermentative *Lactobacilli* were detected in a high proportion in Motal cheese samples, which is in agreement with the work of (Jokovic et al. 2011) which was carried out on Radan traditional cheese.

Singh and Singh (2014) stated that although biochemical tests are commonly used in diagnosis of *Lactobacillus* spp., but often these tests are unable to distinguish between nearly related species in identical ecological environments, such as ripe cheese. As proof for biochemical tests, molecular approach based on PCR analysis can give reliable identification results. In this study, biphasic approach was carried out for identification and typing the *Lactobacilli* isolates of Motal cheese.

Several *Lactobacilli* including *Lactobacillus plantarum*, *Lactobacillus paracasei*, *Lactobacillus rhamnosus*, *Lactobacillus curvatus* and also *Lactobacillus brevis* species have been isolated from ripened cheddar cheese (Singh and Singh 2014). *Lactobacillus plantarum* and *Lactobacillus brevis* representing the most predominant species in Motal cheese.

*Lactobacillus brevis* showed the highest population (65%) of *Lactobacilli* isolates in Motal cheese, as it is the case for many artisanal dairy products (Naylor and Sharpe 1958; Tzanetakis and Litopoulou-Tzanetaki 1992).

Senbagam et al. (2013) studied the Brevicin production by *Lactobacillus brevis* isolates from goat’s milk and their effect against 19 indicator microorganisms including *Bacillus*, *Listeria*, *Lactobacillus*, *Enterococcus*, *Leuconostoc*, *Pseudomonas, Salmonella*, *Clostridium*, *Pediococcus* and *Staphylococcus aureus.* The results indicated that *Staphylococcus aureus* was the most sensitive to bacteriocin.

In our study, all *Lactobacillus plantarum* strains showed antibacterial activity against three studied indicators. Furthermore, Sankar et al. (2012) observed that *Lactobacillus plantarum* isolates from raw cow milk samples had a powerful antimicrobial activity against a set of indicator microorganisms.

In other studies carried out on the antimicrobial activity of LAB isolates from camel and goat’s milk, several cases of growth inhibitory were reported byLAB isolates against the food-borne pathogens (Nikolic et al. 2008; Yateem et al. 2008; Bunesova et al. 2014).

It is now well known that some LAB species produce antimicrobial substances such as hydrogen peroxide, organic acids, bacteriophages and bacteriocins as natural food preservatives (Mozzi et al. 2015). In this study, the antimicrobial activity of identified *Lactobacilli* isolates was carried out using agar spot and well diffusion methods. In many previous studies, it has been observed that although some strains were exhibited positive antimicrobial activity by spot on the lawn assay, their liquid medium (i.e., CFS) did not illustrate that inhibitory activity. This is probably due to the presence of colony- associated antimicrobial compounds such as the fatty acids, ammonia, hydrogen peroxide, and diacetyl which are also responsible for inhibiting growth of indicator bacteria on the lawn (Lima et al. 2007; Alegría et al. 2010; Al-Otaibi 2012).

Sharing our results, Engelhardt et al. (2015), reported that *Listeria innocua* NCTC 11288 was the most resistant bacterium to supernatant produced by the LAB species.

The presence of plnEF gene in all identified *Lactobacillus plantarum* strains was confirmed, which correspond with the results of Chaalel et al. (2015) based on the observation of the PCR product using specific primers of plnEF in *Lactobacillus plantarum* LbM2a.

*Pln*EF and *pln*JK are categorized as class IIb bacteriocins, whose activity depends on the overall activity of two peptides. Two peptide bacteriocins need both peptides for their activity and both peptides act synergistically. The effect of both peptides is found to be greater than the total activity calculated for the effect of each peptide, separately (Todorov 2009). These bacteriocins have little gene coding sequence similarity to other neighboring plantaricins; any peptide combination other than both synergistic peptides in these bacteriocins resulted in full loss of synergies (Zacharof and Lovitt 2012).

Two polypeptide components of Brevicin 174A include Brevicin 174A-β and 174A-γ, that inhibit the growth of closely related organisms as well as pathogenic bacteria such as *Staphylococcus aureus*, *Streptococcus mutans* and *Listeria monocytogenes* (Noda et al. 2015).

Noda et al. (2015) proposed that gene cluster Brevicin 174A contains two transcriptional regulator genes (*bre*D and *bre*G), in addition to the 5 genes necessary for biosynthesis of bacteriocin class IIb (*breA*, *breB*, *breC*, *breE*, and *breH*). Brevicin 174A gene is located on plasmid harbor in *Lactobacillus brevis* 174A, so the experimental primers were designed based on the nucleotide sequences of *bre*C gene from *Lactobacillus brevis* 925A (Accession No. AB370337) (Noda et al. 2015). This gene cluster is identical with Brevicin 925A of *Lactobacillus brevis* 925A which was previously separated from Kimchi by Wada et al. (2009).

Results of the PCR analysis of nucleotides sequence, using specific primers *pln*EF and *pln*A in *Lactobacillus plantarum* isolates represent 100% homology of amplicons of *pln*A and *pln*EF primers with the structure of related bacteriocins. Moreover, amplicons of *bre*174A primers exhibit 99% homology to Brevicin 174A. Mills et al. (2011) also reported that *Lactobacillus plantarum* LMG P-26358 isolated from a type of French cheese produces a strong bacteriocin class II which has 100% homology to Plantaricin 423 and inhibits the growth of two pathogenic and non-pathogenic species of *Listeria* including *Listeria innocua* and *Listeria monocytogenes*.

## Abbreviations

LAB: Lactic Acid Bacteria
GRAS: Generally Regarded As Safe
PCA: Plate Count Agar
MRS: de Man, Rogosa and Sharpe
VRBA: Violet Red Bile Agar
BP: Baird Parker
TBE: Tris/Borate/EDTA
rRNA: ribosomal RNA
27F: 27 forward
1492R: 1492 reverse
PCR: Polymerase Chain Reaction
Gel doc: Gel Documentation
bp: base pair
BLAST: Basic Local Alignment Search Tool
BHI: Brain Heart Infusion
CFS: Cell-Free Supernatant
